# Temporal Distribution of Elevated-HbA1c Records and a Young-Adult Multiorgan Biomarker Profile in Diabetes: A Retrospective Observational Study

**DOI:** 10.3390/diseases14100379

**Published:** 2026-10-15

**Authors:** Umar Saeed, Muhammad Rehan Uppal, Rizwan Uppal, Zsolt Jenő Szepesváry, Zahra Zahid Piracha

**Affiliations:** 1 Korea University College of Health Sciences, Korea University, Seongbuk-gu, Seoul 02841, Republic of Korea; 2 Institute of Graduate Studies and Research, Cyprus International University, 99258 Nicosia, Cyprus; 3 Islamabad Diagnostic Center (IDC), Islamabad 44000, Pakistan; rehanuppal@idc.net.pk (M.R.U.); rizwanuppal@idc.net.pk (R.U.); 4 International Center of Medical Sciences Research (ICMSR), Islamabad 44000, Pakistan; 5 Faculty of Health, Széchenyi István University, 9026 Győr, Hungary; szepesvary.zsolt.jeno@ga.sze.hu (Z.J.S.); piracha.zahra@gmail.com (Z.Z.P.); 6 Center for Research Development, University of Cyberjaya, Persiaran Bestari, Cyber 11, Cyberjaya 63000, Selangor, Malaysia

**Keywords:** HbA1c, diabetes, albuminuria, non-albuminuric diabetic kidney disease, dyslipidemia, liver enzymes, young adults, retrospective observational study, Pakistan

## Abstract

Background/Objectives: Diabetes-related renal, lipid, and hepatic abnormalities are clinically important, but their distribution may be heterogeneous and is not fully represented by HbA1c alone. We characterized the temporal age and sex composition of elevated-HbA1c records and, separately, the multiorgan biomarker profile of a 2022 young-adult subcohort. Methods: This retrospective observational study analyzed 1160 patient-year records classified as having elevated HbA1c from 2019–2022. These were annual record strata rather than 1160 unique individuals or a cohort followed as participants aged. A separate cross-sectional biomarker subcohort comprised 49 adults aged 18–29 years in 2022, of whom 26 were source-classified as having elevated HbA1c. Annual age and sex distributions were assessed using chi-square tests. Biomarker abnormalities were summarized with biomarker-specific denominators and Wilson 95% confidence intervals; sex comparisons were exploratory and used Fisher’s exact tests, odds ratios, φ coefficients, and Benjamini–Hochberg false-discovery-rate correction. Results: Age composition differed across calendar years (χ^2^[6] = 45.02, *p* < 0.001; Cramér’s V = 0.139), with adults aged ≥60 years accounting for 50.0% of elevated-HbA1c records in 2022. Sex composition also varied (χ^2^[3] = 19.47, *p* < 0.001; Cramér’s V = 0.130). In the young-adult biomarker subcohort, the most frequent abnormalities were total cholesterol (60.0%), triglycerides (53.8%), UACR (45.5%), urea (40.0%), uric acid (40.0%), and ALT (35.3%). Elevated urea showed the largest female-to-male odds ratio (17.50, 95% CI 1.22–250.37; *p* = 0.041), but no sex–biomarker association remained significant after false-discovery-rate correction (minimum q = 0.295). Conclusions: The study demonstrates temporal heterogeneity in elevated-HbA1c record composition and a descriptive young-adult biomarker profile dominated by dyslipidemia, albuminuria, selected renal–metabolic abnormalities, and ALT elevation. Because the archived dataset did not retain the paired continuous HbA1c–biomarker data or older-age biomarker data needed for dose–response or cross-age comparisons, the findings should not be interpreted as establishing an HbA1c-linked multiorgan causal relationship. They support the value of integrated organ-risk assessment and the need for prospective patient-level analyses across HbA1c and age strata.

## 1. Introduction

Diabetes mellitus is a heterogeneous metabolic disorder characterized by persistent hyperglycemia arising from impaired insulin secretion, reduced insulin action, or a combination of both mechanisms. Although glucose dysregulation defines the disease, its clinical burden is driven largely by progressive injury across multiple organ systems, particularly the kidneys, cardiovascular system, liver, peripheral nerves, and microvasculature. Consequently, the biological consequences of diabetes cannot be fully understood through isolated glucose measurements alone. A clinically meaningful evaluation requires consideration of how sustained glycemic exposure is reflected across interconnected renal, hepatic, lipid, and metabolic pathways. The broader diabetes-care framework also includes prevention and surveillance for neurologic, retinal, foot, and other comorbid complications [1,2].

Glycated hemoglobin (HbA1c) is the most widely used indicator of average glycemic exposure over the preceding approximately 8–12 weeks. Compared with a single plasma glucose measurement, HbA1c is less affected by short-term fasting status, acute dietary intake, and daily glucose fluctuations, making it valuable for diabetes diagnosis, longitudinal monitoring, and therapeutic decision-making [3,4]. An HbA1c concentration of ≥6.5% (48 mmol/mol), when confirmed in the absence of unequivocal hyperglycemia, is an established diagnostic criterion for diabetes. However, a diagnostic threshold should not be interpreted as a universal definition of inadequate glycemic control among individuals with established disease. For many non-pregnant adults, an HbA1c target below 7.0% is commonly recommended, but appropriate treatment goals must be individualized according to age, diabetes duration, comorbidities, life expectancy, treatment burden, and risk of hypoglycemia [5,6]. HbA1c should therefore be interpreted as a continuous marker of glycemic exposure rather than as a simple binary indicator of normal or abnormal control. Contemporary guidance also emphasizes use of complementary glucose-monitoring technologies when clinically appropriate [7,8].

Sustained hyperglycemia is associated with microvascular and macrovascular complications, but organ-level abnormalities do not develop uniformly across all people with diabetes. Kidney, lipid, and hepatic biomarkers may therefore provide complementary information to HbA1c, particularly when interpreted as distinct clinical domains rather than as interchangeable markers of a single process.

Renal assessment requires both albuminuria and filtration measures. An increased urine albumin-to-creatinine ratio (UACR) can indicate glomerular injury, whereas serum creatinine and the estimated glomerular filtration rate (eGFR) reflect filtration. Importantly, diabetic kidney disease is heterogeneous: a reduced eGFR can occur despite a normal UACR, so an albuminuria-centered interpretation may miss a non-albuminuric phenotype. Urea and uric acid may add metabolic or renal context but are nonspecific. Lipid abnormalities and aminotransferase elevations likewise provide clinically useful signals, although neither dyslipidemia nor abnormal liver enzymes establish a specific organ diagnosis without additional evaluation [9,10,11,12,13,14,15].

Although HbA1c is central to diabetes management, it is not an infallible representation of glycemia. Its interpretation may be distorted by conditions that alter erythrocyte lifespan, hemoglobin structure, or red-cell production, including iron-deficiency anemia, hemolysis, hemoglobinopathies, recent blood loss or transfusion, pregnancy, advanced kidney disease, and erythropoietin treatment [16]. Moreover, two individuals with comparable HbA1c values may differ substantially in glycemic variability, postprandial excursions, time within the target glucose range, and cumulative exposure to metabolic risk factors. HbA1c should therefore be interpreted alongside clinical characteristics and complementary biochemical indicators rather than being used as a solitary surrogate for systemic diabetic injury [8].

Routine renal, lipid, and hepatic tests are inexpensive and widely available, which makes multidomain assessment attractive in settings where advanced imaging or molecular testing is not routinely accessible. Current diabetes and kidney-disease guidance emphasizes integrated risk assessment rather than reliance on a single laboratory marker [10,11,15,17,18]. However, the added value of such testing depends on transparent reporting of who was tested, which measurements were available, and whether organ biomarkers can actually be linked to HbA1c at the individual-patient level.

The present dataset offers two complementary but distinct views of diabetes care: a four-year record-level description of elevated HbA1c by age and sex, and a detailed 2022 biomarker profile available only in a young-adult subcohort. The study does not attempt to re-establish the known fact that diabetes can affect multiple organs; rather, its added value lies in describing the local temporal distribution of elevated-HbA1c records and the pattern and analytical coverage of routinely available renal, lipid, hepatic, and protein biomarkers in an underrepresented Pakistani clinical setting.

Accordingly, this study had three objectives: (1) to describe how elevated-HbA1c patient-year records were distributed across age and sex strata from 2019 to 2022; (2) to characterize the frequency of renal, lipid, hepatic, and serum-protein abnormalities in a separate 2022 subcohort of adults aged 18–29 years; and (3) to explore sex-associated differences within that young-adult biomarker subcohort. The age categories represent different record strata rather than the same participants followed as they aged. Because the archived biomarker dataset did not retain the patient-level continuous values needed for HbA1c-category comparisons or correlation/regression analyses, those analyses were not undertaken in the revised study.

## 2. Materials and Methods

### 2.1. Study Design and Data Structure

This retrospective observational study was conducted at the International Center of Medical Sciences Research (ICMSR), Islamabad, Pakistan, using routinely generated clinical and laboratory information from 2019–2022. The revision distinguishes two analytically separate datasets. The first was a repeated annual cross-sectional summary of 1160 patient-year records classified in the source dataset as having elevated HbA1c. The unit of analysis for this component was a patient-year record, not a unique patient. A patient could therefore contribute records in more than one calendar year, and the available annual summary did not permit reconstruction of within-person HbA1c trajectories.

The second dataset was a separate cross-sectional 2022 biomarker subcohort comprising 49 adults aged 18–29 years who had an HbA1c assessment and at least one available renal, lipid, hepatic, or serum-protein measurement. This subcohort was analyzed at the individual level and was not treated as a longitudinal continuation of the annual record dataset.

### 2.2. Annual Elevated-HbA1c Record Dataset

Annual elevated-HbA1c records were summarized by calendar year, recorded biological sex, and age at the time of testing. Age was classified into three mutually exclusive groups: 18–29 years, 30–59 years, and ≥60 years. These categories represent different records from individuals who belonged to those age bands at the time of testing; they do not represent the same participants followed prospectively as they moved from one age group to another.

The archived annual dataset contained counts of source-classified elevated-HbA1c records but did not contain the total number of people tested in every year-by-age-by-sex stratum. Accordingly, annual results are interpreted as the composition of observed elevated-HbA1c records and not as prevalence, incidence, or age-specific risk. The archived summary also did not retain a complete patient-level numerical HbA1c series for all 1160 records; mean HbA1c and within-patient change were therefore not estimated.

### 2.3. The 2022 Young-Adult Biomarker Subcohort

The 2022 biomarker subcohort included 49 adults aged 18–29 years (28 males and 21 females). Within the source dataset, 26 of 49 participants (53.1%) were classified as having elevated HbA1c: 16 of 28 males and 10 of 21 females. Detailed biomarker availability was incomplete and varied by analyte; individual biomarkers were available for only 11–17 participants.

Equivalent detailed biomarker-level data were not available in the archived analytical dataset for the 30–59-year and ≥60-year strata. For this reason, renal, hepatic, and metabolic biomarkers were not compared across age groups. Likewise, the archived biomarker export did not preserve the complete paired continuous HbA1c and biomarker values, or the biomarker-by-HbA1c cross-tabulations, required for valid comparisons across HbA1c categories. No values were imputed or reconstructed for these unavailable analyses.

### 2.4. Biomarker Domains and Abnormality Classification

The renal/metabolic domain comprised the UACR, serum urea, a combined creatinine/eGFR classification, and serum uric acid. The lipid domain comprised total cholesterol, triglycerides, LDL-C, HDL-C, and VLDL-C. The hepatic domain comprised total bilirubin, alkaline phosphatase, AST, ALT, and GGT. Serum albumin and total protein were assessed as additional protein-related markers.

Where the archived numerical value and applicable decision limit were available, UACR ≥ 30 mg/g was considered abnormal. Lipid abnormalities were defined as total cholesterol ≥ 200 mg/dL, triglycerides ≥ 150 mg/dL, LDL-C ≥ 100 mg/dL, HDL-C < 40 mg/dL in males or <50 mg/dL in females, with VLDL-C interpreted according to the source laboratory classification. Urea, uric acid, creatinine/eGFR, bilirubin, alkaline phosphatase, AST, ALT, GGT, albumin, and total protein were classified using the contemporaneous laboratory reference interval represented in the verified source summary. Missing measurements were treated as missing and never classified as normal.

Because the source summary retained creatinine and the eGFR as a combined normal/abnormal category rather than separate numerical values, this study cannot distinguish isolated creatinine elevation from a reduced eGFR, cannot identify eGFR-defined non-albuminuric diabetic kidney disease, and cannot calculate the serum uric acid-to-creatinine ratio.

### 2.5. Rationale for Sex-Based Analyses

Sex-based analyses were prespecified as exploratory because several laboratory measures have sex-dependent distributions or decision limits and because biological sex can influence lipid, renal, and hepatic biomarker patterns. The purpose was to describe whether abnormality frequencies differed between males and females within the young-adult subcohort, not to infer a causal biological mechanism. Given the small biomarker-specific samples, sex findings were interpreted using effect sizes, confidence intervals, and multiplicity correction rather than nominal *p*-values alone.

### 2.6. Statistical Analysis

Categorical variables were summarized as counts and percentages. Biomarker abnormality proportions were calculated using the number of participants with an available result for that biomarker as the denominator, and Wilson 95% confidence intervals were used for the overall abnormality proportions.

Differences in age-group composition across the four calendar years were evaluated using the chi-square test, with Cramér’s V as the effect-size measure. Calendar-year differences in sex composition and age-by-sex heterogeneity were also assessed using chi-square tests. In the 49-person biomarker subcohort, the association between source-classified elevated HbA1c and sex was assessed using Fisher’s exact test.

Exploratory sex–biomarker comparisons used two-sided Fisher’s exact tests, female-to-male odds ratios, and φ coefficients. For 2 × 2 tables containing a zero cell, a Haldane–Anscombe continuity correction of 0.5 was used for the odds-ratio confidence interval; Fisher’s exact *p*-value was calculated from the uncorrected table. The Benjamini–Hochberg procedure was applied across the nine reported sex–biomarker comparisons. A two-sided *p* value < 0.05 was considered nominally significant, while false-discovery-rate-adjusted q values were used to judge multiplicity-adjusted significance.

Correlation analysis, multiple linear regression, spline modeling, mixed-effects modeling, and HbA1c-category comparisons are not reported because the archived summary dataset did not contain the paired patient-level continuous measurements required to perform those analyses validly. 

### 2.7. Ethical Approval and Confidentiality

The study protocol was approved by the Institutional Review Board and Research Ethics Committee of ICMSR, Islamabad, Pakistan (protocol code ICMSR/IRB/17862019; approval year 2019), and was conducted in accordance with the Declaration of Helsinki. The exact day and month of approval are not available in the archived IRB documentation retained for this retrospective study. The Institutional Review Board authorized retrospective analysis of de-identified clinical and laboratory records under a minimal-risk protocol. For the 2022 in-person biochemical assessment, a written participant consent form was provided to each participant and signed before inclusion. Patient identifiers were replaced with coded study numbers before analysis.

## 3. Results

### 3.1. Study Datasets, Sample Size, and Analytical Scope

A total of 1160 patient-year records classified as having elevated HbA1c were available for the 2019–2022 annual analysis. These 1160 observations are patient-year records rather than 1160 unique patients. Of the records, 652 were from males (56.2%) and 508 from females (43.8%); 165 records (14.2%) were from adults aged 18–29 years, 510 (44.0%) from adults aged 30–59 years, and 485 (41.8%) from adults aged ≥60 years.

The age strata are cross-sectional categories defined at the time of each record. They do not represent the same individuals followed as they aged. Because stratum-specific testing denominators were unavailable, the annual analyses describe the composition of observed elevated-HbA1c records rather than prevalence, incidence, or longitudinal disease progression.

The separate 2022 biomarker subcohort included 49 adults aged 18–29 years (28 males and 21 females). Twenty-six of 49 participants (53.1%) were source-classified as having elevated HbA1c: 16 of 28 males (57.1%) and 10 of 21 females (47.6%). This proportion did not differ significantly by sex (Fisher’s exact *p* = 0.572; female-to-male OR 0.68; risk ratio 0.83, 95% CI 0.48–1.45; φ = −0.094). The 49-person subcohort should not be interpreted as a subset of the 1160 annual records selected on the basis of age alone; it was a distinct 2022 analytical sample with more detailed biochemical information.

### 3.2. Temporal Redistribution of Elevated-HbA1c Records

The annual number of elevated-HbA1c records was 300 in 2019, 289 in 2020, 309 in 2021, and 262 in 2022. Relative to the preceding year, this represented a 3.7% reduction in 2020, a 6.9% increase in 2021, and a 15.2% reduction in 2022.

The age composition differed significantly across calendar years (χ^2^[6] = 45.02, *p* < 0.001; Cramér’s V = 0.139), indicating a statistically supported, although modest, temporal redistribution among age groups.

In 2019, adults aged 30–59 years constituted the largest group, contributing 131 of 300 records (43.7%). Adults aged ≥60 years contributed 120 records (40.0%), while those aged 18–29 years contributed 49 records (16.3%).

The concentration in middle age became more pronounced in 2020, when adults aged 30–59 years accounted for 145 of 289 records (50.2%). Adults aged ≥60 years accounted for 119 records (41.2%), whereas adults aged 18–29 years contributed 25 records (8.7%).

A similar pattern persisted in 2021. The 30–59-year group contributed 159 of 309 records (51.5%), compared with 115 records among adults aged ≥60 years (37.2%) and 35 records among adults aged 18–29 years (11.3%).

The age distribution changed substantially in 2022. Adults aged ≥60 years accounted for 131 of 262 records, representing exactly 50.0% of the annual total. The 30–59-year group declined to 75 records (28.6%), while adults aged 18–29 years increased to 56 records (21.4%).

Between 2021 and 2022, records among adults aged 30–59 years decreased from 159 to 75, corresponding to a 52.8% reduction. In contrast, records among adults aged ≥60 years increased by 13.9%, and records among adults aged 18–29 years increased by 60.0%.

**Figure 1:**
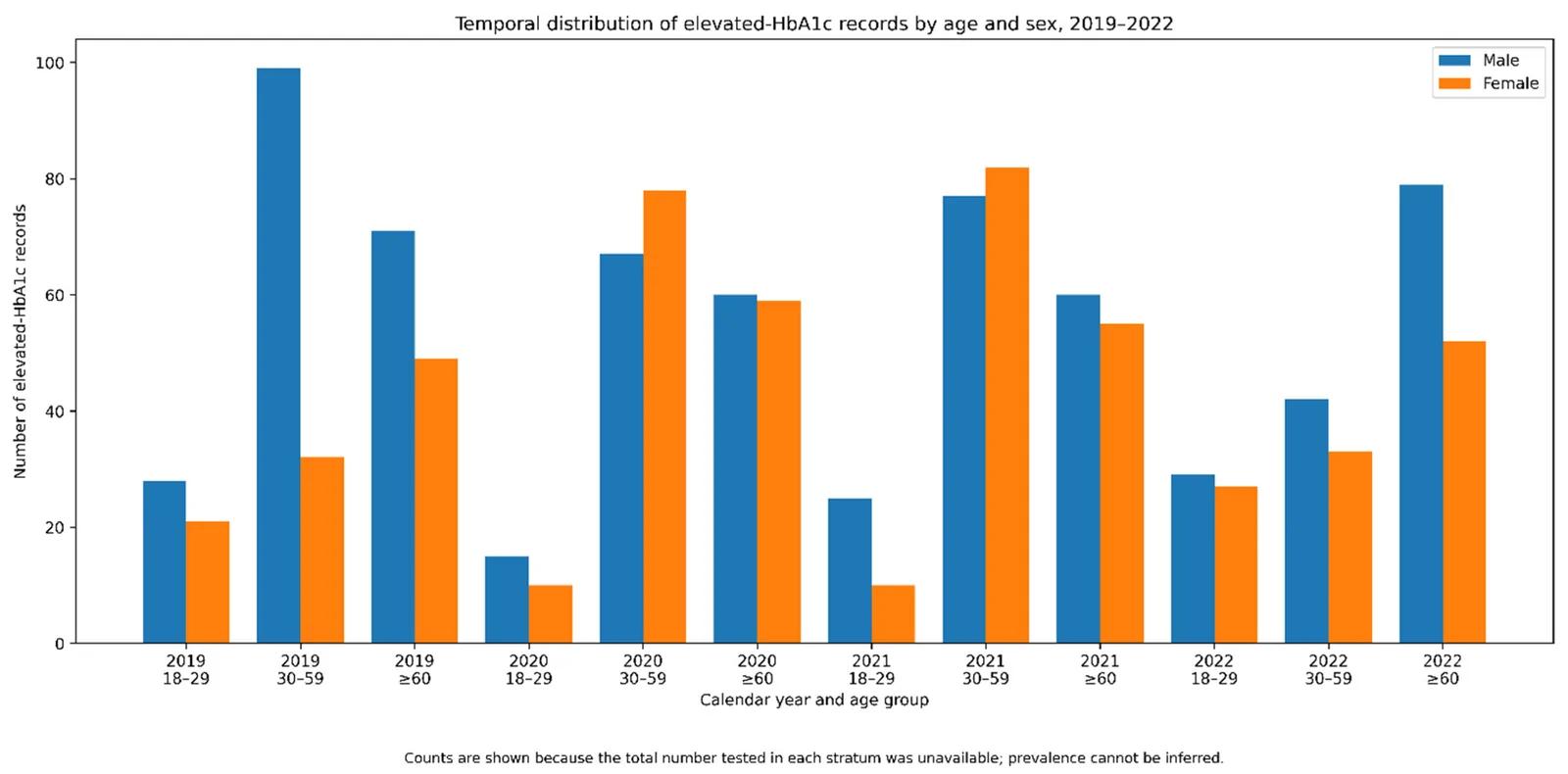
Temporal distribution of elevated-HbA1c records by age and sex, 2019–2022. Grouped bars show male and female record counts within the 18–29-year, 30–59-year, and ≥60-year age groups. The figure demonstrates a middle-aged concentration during 2020–2021 and a shift toward older adults in 2022. Counts are presented because the total number tested in each stratum was unavailable; prevalence cannot be inferred.

The dominant age contribution therefore shifted from middle-aged adults during 2019–2021 toward older adults in 2022, accompanied by a renewed contribution from young adults. Because testing denominators were unavailable, these changes should not be interpreted as changes in age-specific prevalence. Annual age-by-sex record counts are given in Table 1 and illustrated in Figure 1.

### 3.3. Sex Distribution Across Calendar Years

The sex composition of elevated-HbA1c records also differed significantly across calendar years (χ^2^[3] = 19.47, *p* < 0.001; Cramér’s V = 0.130).

In 2019, males accounted for 198 of 300 records (66.0%; 95% CI 60.5–71.1%), compared with 102 female records (34.0%). The largest sex difference occurred in the 30–59-year group, with 99 male records and 32 female records.

The overall distribution became nearly balanced in 2020, comprising 142 male records (49.1%) and 147 female records (50.9%). Within the 30–59-year group, females contributed 78 records compared with 67 among males. The ≥60-year group was almost equally represented, with 60 male and 59 female records.

In 2021, males accounted for 162 of 309 records (52.4%), while females contributed 147 records (47.6%). Females slightly outnumbered males within the 30–59-year group, with 82 compared with 77 records.

Male predominance re-emerged in 2022. Males contributed 150 of 262 records (57.3%), compared with 112 records among females (42.7%). The greatest absolute difference occurred in adults aged ≥60 years, with 79 male and 52 female records.

Age-by-sex heterogeneity was observed in 2019 (χ^2^[2] = 9.56, *p* = 0.008; Cramér’s V = 0.179) and was weaker in 2021 (χ^2^[2] = 6.09, *p* = 0.048; Cramér’s V = 0.140). No significant age-by-sex heterogeneity was detected in 2020 or 2022.

When all four years were pooled, the age distribution was comparable between males and females (χ^2^[2] = 0.53, *p* = 0.769; Cramér’s V = 0.021). This indicates that the observed sex differences were calendar-year-dependent rather than reflecting a persistent imbalance across age groups.

### 3.4. Young-Adult Biomarker Coverage and Abnormality Profile

Detailed biochemical testing was incomplete: depending on the analyte, only 11–17 of the 49 young adults had an available result. Because the archived analytical summary did not retain paired continuous HbA1c–biomarker values or biomarker-by-HbA1c cross-tabulations, biomarker results cannot be validly compared across HbA1c categories in this dataset. The descriptive abnormality profile is therefore reported using biomarker-specific available-case denominators.

The most frequent abnormalities were elevated total cholesterol in 9 of 15 participants (60.0%; 95% CI 35.7–80.2%), elevated triglycerides in 7 of 13 (53.8%; 95% CI 29.1–76.8%), an abnormal UACR in 5 of 11 (45.5%; 95% CI 21.3–72.0%), elevated urea in 6 of 15 (40.0%; 95% CI 19.8–64.3%), elevated uric acid in 6 of 15 (40.0%; 95% CI 19.8–64.3%), and elevated ALT in 6 of 17 (35.3%; 95% CI 17.3–58.7%).

Less frequent abnormalities included AST elevation in 2 of 13 participants (15.4%), LDL-C elevation in 2 of 13 (15.4%), and total bilirubin elevation in 1 of 11 (9.1%). No abnormal results were recorded among those tested for the combined creatinine/eGFR classification, alkaline phosphatase, GGT, serum albumin, total protein, HDL-C, or VLDL-C.

These data provide counts and proportions of laboratory abnormalities, not mean or median analyte concentrations. Continuous HbA1c, liver-enzyme, urea, creatinine, and related measurements were not recoverable as complete paired patient-level values from the archived analytical summary and were therefore not reconstructed. The available-case abnormality counts by sex are summarized in Table 2.

**Figure 2:**
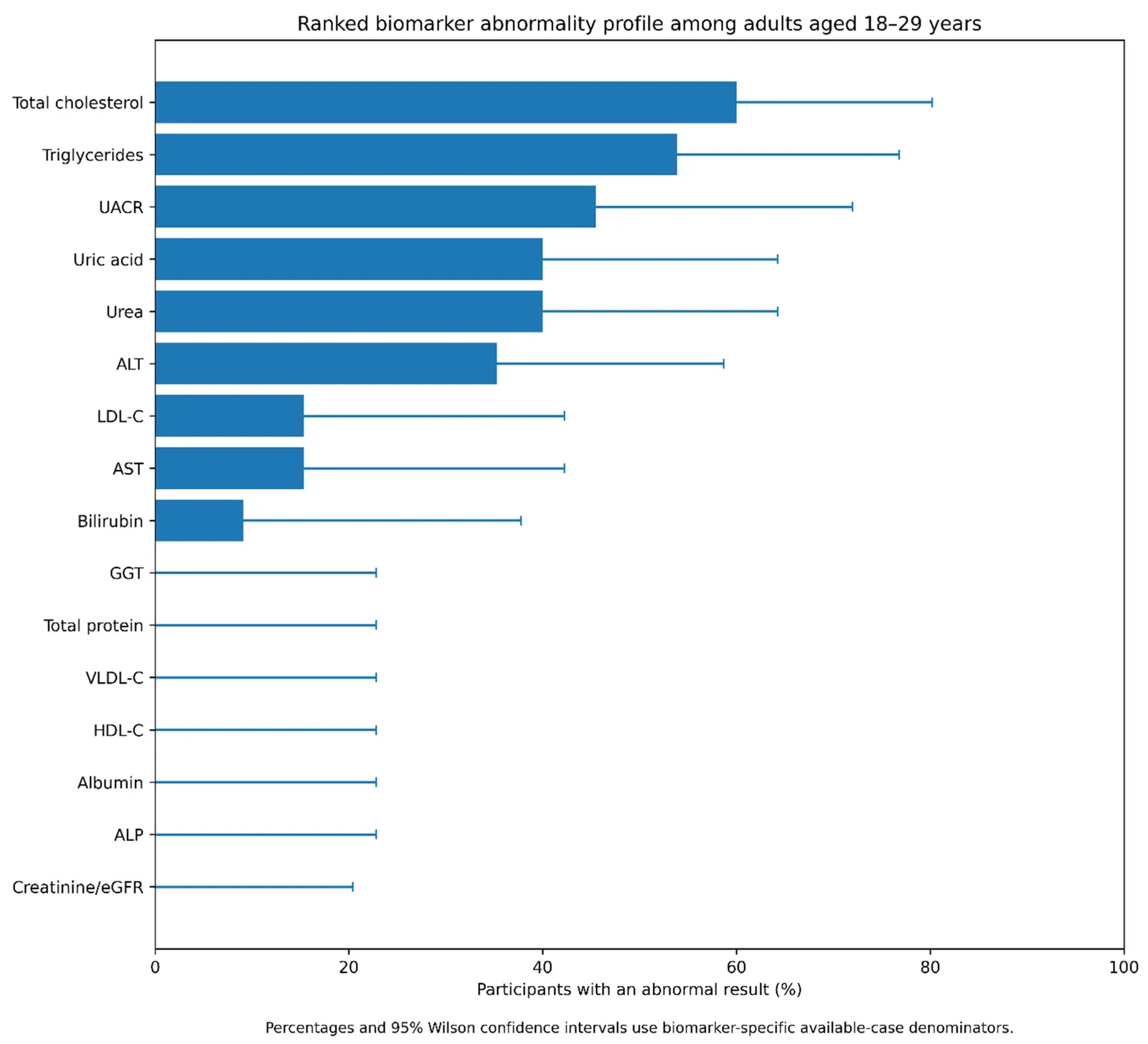
Ranked biomarker-abnormality profile in the 2022 young-adult biomarker subcohort. Bars show biomarker-specific abnormality percentages with 95% Wilson confidence intervals. Percentages use biomarker-specific available-case denominators and should not be interpreted as HbA1c-category-specific rates.

The ranked abnormality profile and associated 95% confidence intervals are presented in Figure 2.

### 3.5. Sex-Associated Biomarker Patterns

Binary sex–biomarker associations were evaluated using Fisher’s exact tests, odds ratios, and φ coefficients. Female sex was coded as the comparison group; therefore, an OR above 1 indicated more frequent abnormality among females, while an OR below 1 indicated more frequent abnormality among males.

The strongest female-directed association was observed for elevated serum urea. Elevated urea occurred in five of seven females (71.4%) compared with one of eight males (12.5%), producing an OR of 17.50 (95% CI 1.22–250.37; Fisher’s exact *p* = 0.041; φ = 0.600). After Benjamini–Hochberg correction across the nine exploratory sex–biomarker comparisons, this association was not statistically significant (q = 0.295).

ALT elevation was also more frequent among females, occurring in five of nine females (55.6%) and one of eight males (12.5%). The corresponding OR was 8.75 (95% CI 0.74–103.83; *p* = 0.131; φ = 0.450; q = 0.295).

Elevated uric acid was present in four of seven females (57.1%) and two of eight males (25.0%), yielding an OR of 4.00 (95% CI 0.45–35.79; *p* = 0.315; φ = 0.327).

The strongest male-directed association involved triglyceride elevation. Elevated triglycerides occurred in five of six males (83.3%) and two of seven females (28.6%), corresponding to a female-to-male OR of 0.08 (95% CI 0.01–1.19; *p* = 0.103; φ = −0.548; q = 0.295).

Total cholesterol elevation was observed in six of seven males (85.7%) compared with three of eight females (37.5%), producing a female-to-male OR of 0.10 (95% CI 0.01–1.29; *p* = 0.119; φ = −0.491; q = 0.295).

**Figure 3:**
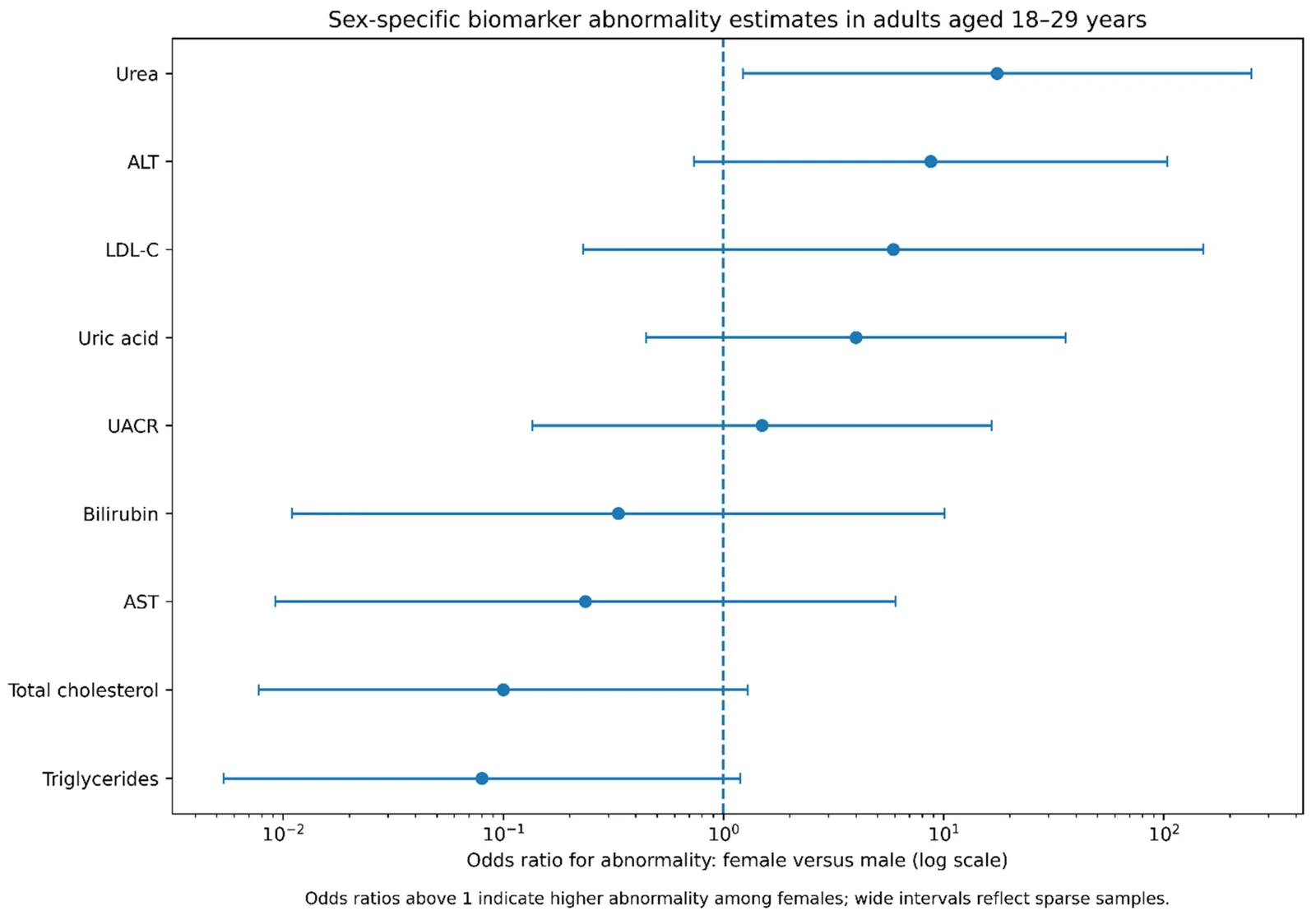
Exploratory sex-specific biomarker abnormality estimates in adults aged 18–29 years. The forest plot presents female-to-male odds ratios on a logarithmic scale. Estimates above 1 indicate more frequent abnormality among females and estimates below 1 more frequent abnormality among males. Wide confidence intervals reflect sparse biomarker-specific samples; none of the associations remained significant after false-discovery-rate correction.

The complete sex-effect estimates are presented in Figure 3 and Table 3. No sex–biomarker association remained significant after false-discovery-rate correction; the minimum q value was 0.295. The wide confidence intervals reflect sparse biomarker-specific samples and make these comparisons hypothesis-generating only.

For zero-cell tables, odds ratios and 95% confidence intervals use a Haldane–Anscombe correction; Fisher’s exact *p*-values use the uncorrected 2 × 2 table. FDR q-values were calculated using the Benjamini–Hochberg procedure across the nine reported sex–biomarker comparisons. Positive φ values indicate female-directed associations and negative values male-directed associations.

**Figure 4:**
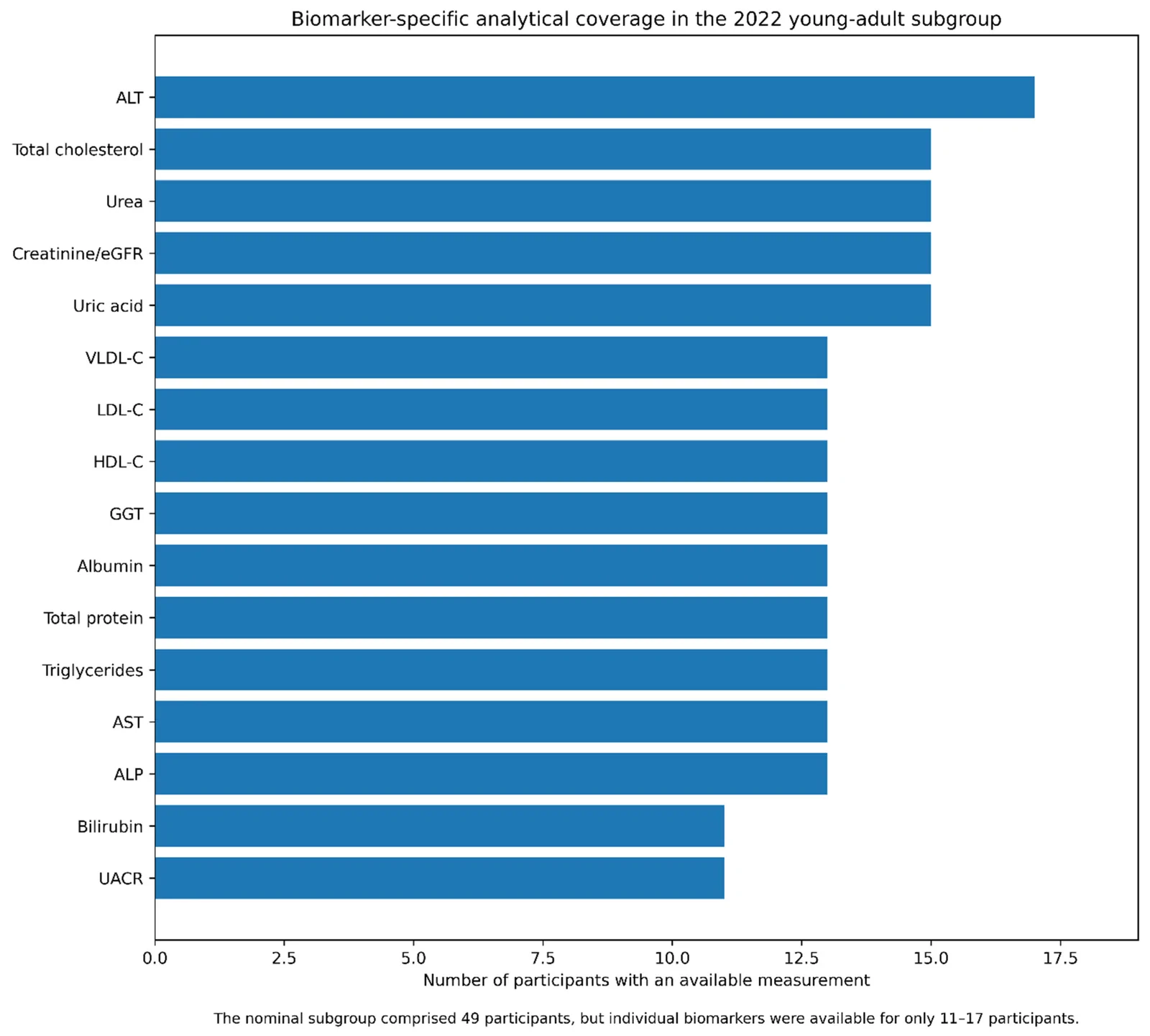
Biomarker-specific analytical coverage in the 2022 young-adult subcohort. Bars show the number of participants contributing an available result for each biomarker. Although the subcohort comprised 49 participants, individual biomarker analyses were based on only 11–17 available results.

Biochemical measurement availability varied considerably. Individual biomarkers were available for only 11–17 of the 49 young adults. Consequently, every biomarker percentage was calculated using its own available-case denominator, and missing measurements were not classified as normal. The biomarker-specific analytical coverage is presented in Figure 4. As shown in Figure 4, the small and unequal coverage limits cross-analyte comparisons.

### 3.6. Renal and Renal–Metabolic Biomarker Profile

The renal profile was characterized by an abnormal UACR in 5 of 11 tested participants (45.5%) and elevated urea and uric acid in 6 of 15 each (40.0%). These findings describe the frequency of abnormal tests among those measured; they do not establish chronic kidney disease, diabetic kidney disease, or a temporal sequence of renal injury.

An abnormal UACR was present in two of five males (40.0%) and three of six females (50.0%). The female-to-male OR was 1.50 (95% CI 0.14–16.54; Fisher’s exact *p* = 1.000), indicating substantial imprecision.

Elevated serum urea showed the largest nominal sex difference, occurring in five of seven females and one of eight males, but this association did not remain significant after FDR correction. Urea is a nonspecific marker and can vary with hydration, protein intake, catabolic state, hepatic urea production, and renal perfusion.

Elevated uric acid was observed in 40.0% of tested participants and was more frequent descriptively among females. Uric acid is interpreted here as an exploratory renal–metabolic marker rather than evidence of urate-mediated kidney disease.

No abnormality was recorded in the combined creatinine/eGFR classification among the 15 participants with an available result. Because the source summary did not retain serum creatinine and the eGFR as separate numerical variables, this finding cannot be used to quantify filtration, identify a reduced eGFR with a normal UACR, or exclude non-albuminuric diabetic kidney disease.

Accordingly, the renal results should be viewed as a limited descriptive profile rather than an albuminuria-defined renal phenotype. Identification of albuminuric and non-albuminuric diabetic kidney disease requires separate UACR and eGFR measurements, appropriate chronicity, and patient-level pairing.

### 3.7. Lipid Phenotype

The lipid domain contained the highest burden of observed abnormalities.

Elevated total cholesterol affected 60.0% of examined participants, while elevated triglycerides affected 53.8%. Both abnormalities were concentrated descriptively among males.

Among males, total cholesterol and triglycerides were abnormal in 85.7% and 83.3%, respectively. Among females, the corresponding proportions were 37.5% and 28.6%.

LDL-C elevation was considerably less frequent, affecting 15.4% overall and occurring only among the examined females. No low HDL-C or elevated VLDL-C result was recorded.

The corrected profile therefore indicates a total-cholesterol and triglyceride-predominant phenotype, rather than generalized disruption of all measured lipid fractions.

### 3.8. Hepatic Biochemical Phenotype

The hepatic profile was focal rather than generalized.

ALT was the most frequently abnormal hepatic marker, affecting 35.3% of participants with an available result. ALT elevation was concentrated descriptively among females, occurring in 55.6% of examined females compared with 12.5% of males.

AST elevation was present in 15.4% overall and occurred only among the examined males. Total bilirubin elevation was uncommon, affecting one male participant.

No GGT or alkaline phosphatase abnormality was recorded. These results directly contradict the previous assertion of universal GGT elevation.

The observed pattern is therefore best characterized as a selective aminotransferase phenotype, driven primarily by ALT elevation in a subset of females and less frequent AST elevation among males. It cannot be interpreted as a diagnosis of metabolic dysfunction-associated steatotic liver disease or another specific hepatic disorder.

### 3.9. Serum Protein Profile

Serum albumin and total protein were normal in all participants with available measurements.

The findings provide no evidence of elevated serum protein concentrations and do not support interpretations involving excessive hepatic protein synthesis, dehydration, hemoconcentration, or protein retention.

### 3.10. Summary of Descriptive Organ-Domain Findings

The young-adult subcohort showed a heterogeneous distribution of laboratory abnormalities across lipid, renal/renal–metabolic, and hepatic domains. Because participant-level cross-domain pairing was not retained in the archived summary, the data do not demonstrate coordinated multisystem abnormalities within the same individuals.

The most frequent abnormalities were in total cholesterol and triglycerides, followed by the UACR, urea, uric acid, and ALT. These frequencies are descriptive and reflect selective testing with biomarker-specific denominators.

Exploratory sex comparisons suggested more frequent total-cholesterol and triglyceride abnormalities among tested males and more frequent urea, uric-acid, and ALT abnormalities among tested females. None of these associations remained significant after false-discovery-rate correction.

No abnormality was recorded among those tested for the combined creatinine/eGFR classification, GGT, alkaline phosphatase, serum albumin, total protein, HDL-C, or VLDL-C; these zero-event findings are based on small denominators and should not be generalized beyond the tested subcohort.

The principal inferential boundary is therefore clear: the study can describe annual elevated-HbA1c record composition and the frequency of biomarker abnormalities in the young-adult subcohort, but it cannot establish an HbA1c dose–response relationship, compare organ biomarkers across HbA1c categories, compare organ biomarkers across age groups, or calculate a patient-level multisystem burden score.

Four findings summarize the revised analysis.

First, the age composition of elevated-HbA1c records changed significantly between 2019 and 2022. Middle-aged adults represented approximately half of the recorded burden during 2020 and 2021, whereas adults aged ≥60 years represented half of all records in 2022.

Second, the sex composition of elevated-HbA1c records also varied by year; these are record-composition differences and not sex-specific prevalence estimates.

Third, the most frequent abnormalities in the 49-person young-adult biomarker subcohort were total cholesterol, triglycerides, the UACR, urea, uric acid, and ALT.

Fourth, exploratory sex-effect estimates were imprecise and none remained significant after multiplicity correction, so they should be treated as hypothesis-generating rather than as evidence of sex-specific pathophysiology.

Overall, the data support a descriptive picture of heterogeneous laboratory abnormalities in young adults undergoing diabetes-related assessment; they do not demonstrate that those abnormalities were caused by, or increased monotonically with, HbA1c.

## 4. Discussion

### 4.1. Principal Findings

The revised analysis separates two questions that were previously blended: the temporal composition of elevated-HbA1c records across 2019–2022 and the descriptive multiorgan biomarker profile of a separate 2022 young-adult subcohort. The annual analysis showed a statistically supported but modest redistribution of elevated-HbA1c records across age and sex strata. The biomarker subcohort showed frequent total-cholesterol, triglyceride, UACR, urea, uric-acid, and ALT abnormalities, but the available data do not establish that these abnormalities increased with HbA1c or co-occurred within the same individuals.

This distinction is important because the 1160 observations are patient-year records rather than 1160 unique people, whereas the 49-person biochemical sample is an individual-level cross-sectional subcohort. The study therefore should not be interpreted as a four-year prospective cohort of the same participants or as a longitudinal analysis of people aging from one category into another.

### 4.2. Age-Group and HbA1c Comparisons: What the Available Data Can and Cannot Address

The age categories in the annual analysis represent different record strata defined at the time of testing. Across all four years, age-group composition differed significantly, and by 2022 adults aged ≥60 years accounted for half of the elevated-HbA1c records. This finding is informative about the composition of the observed clinical workload, but the absence of the total number tested in each stratum prevents interpretation as age-specific prevalence or risk.

The reviewer-suggested comparison of renal, hepatic, and metabolic measurements across age groups and HbA1c categories would be more informative than abnormality percentages alone. We agree with that analytical principle. However, the archived biomarker dataset available for this study contains detailed biochemical information only for the 18–29-year subgroup and does not retain the complete paired continuous HbA1c–biomarker values or biomarker-by-HbA1c cross-tabulations required for a valid category-based analysis. Performing those comparisons retrospectively would therefore require unsupported reconstruction or imputation. The revised manuscript instead narrows its claims to the analyses that the verified data can support and makes this limitation explicit.

### 4.3. Renal Findings, Non-Albuminuric DKD, and Uric-Acid Interpretation

An abnormal UACR was present in 45.5% of tested young adults, while elevated urea and uric acid were each present in 40.0%. No abnormal result was recorded in the combined creatinine/eGFR category among 15 tested participants. These observations should not be interpreted as showing that albuminuria necessarily preceded loss of filtration because the measurements were incompletely paired and creatinine/eGFR was preserved only as a combined categorical variable.

Renal involvement in diabetes is heterogeneous and cannot be reduced to albuminuria alone. Non-albuminuric diabetic kidney disease (NA-DKD) is characterized by reduced kidney function despite a normal UACR; consequently, a normal UACR does not exclude clinically important renal vulnerability. The present dataset cannot identify NA-DKD because separate numerical eGFR values were not retained. This limitation strengthens the need to interpret the UACR jointly with the eGFR rather than to treat albuminuria as the sole renal marker. The recent guideline and review literature similarly supports joint interpretation of the UACR and eGFR and recognizes non-albuminuric DKD as a clinically relevant phenotype [9,10,11,12,13,14,15].

Recent work by Di Marco et al. further illustrates this heterogeneity. In adults with type 2 diabetes, the serum uric acid-to-creatinine ratio (SUA/sCr) differed across DKD phenotypes and was lower in NA-DKD; a lower SUA/sCr was also associated with markers of tubular injury and subclinical vascular damage [19]. Our study includes uric-acid abnormality data but cannot calculate the SUA/sCr because the archived summary does not contain separate continuous serum-creatinine values. Future analyses should preserve serum uric acid, serum creatinine, the eGFR, the UACR, and tubular/vascular markers at the patient level so that albuminuric and non-albuminuric phenotypes can be evaluated directly.

### 4.4. Lipid and Hepatic Findings

The most frequent abnormalities in the young-adult biomarker subcohort were elevated total cholesterol (60.0%) and triglycerides (53.8%). LDL-C elevation was less common, and no low HDL-C or elevated VLDL-C result was recorded among those tested. This pattern supports a descriptive triglyceride/total-cholesterol burden in the tested subgroup but does not establish an increased atherogenic particle number, remnant cholesterol, or apolipoprotein-B burden because those measurements were unavailable [20,21,22,23,24].

The hepatic profile was selective. ALT was elevated in 35.3% of tested participants, whereas AST elevation was less frequent and no GGT or alkaline phosphatase abnormality was recorded. These findings should be interpreted as biochemical abnormalities requiring clinical context rather than as a diagnosis of metabolic dysfunction-associated steatotic liver disease or another specific hepatic disorder [25,26,27,28,29,30,31,32]. 

### 4.5. Rationale and Interpretation of Sex-Based Analyses

Sex-stratified analyses were retained because some laboratory decision limits are sex-specific and because sex can influence the distribution of lipid, renal, and hepatic biomarkers. In the young-adult subcohort, elevated urea and ALT were more frequent descriptively among females, whereas total cholesterol and triglycerides were more frequent among males. However, all estimates were based on small biomarker-specific samples and none remained significant after Benjamini–Hochberg correction. These findings should therefore be regarded as exploratory effect-size estimates rather than evidence of a reproducible sex-specific disease phenotype.

### 4.6. Novel Contribution and Clinical Added Value

The novelty of this study is not the general observation that diabetes can be accompanied by renal, lipid, or hepatic abnormalities; that relationship is already well established. The added value is more specific. First, the study provides a four-year description of how elevated-HbA1c records were distributed by age and sex in a Pakistani clinical setting. Second, it documents a multidomain biomarker-abnormality profile in a young-adult subcohort using explicit available-case denominators, thereby showing both the observed pattern and the substantial incompleteness of routine biochemical testing. Third, the revised analysis demonstrates that the organ-domain pattern is heterogeneous rather than uniform and that stronger causal or HbA1c-linked interpretations are not justified without paired patient-level data.

Clinically, these results reinforce the importance of not viewing HbA1c in isolation. When organ-risk assessment is indicated, the UACR and eGFR should be interpreted together, lipid abnormalities should be assessed within overall cardiovascular risk, and persistent liver-enzyme abnormalities should be evaluated in the appropriate hepatic context. The present data support this integrated surveillance concept descriptively; they do not establish a new treatment algorithm or prove that elevated HbA1c itself triggers each organ abnormality. This integrated approach is consistent with contemporary diabetes, CKD, cardiovascular-risk, lipid, and MASLD guidance [10,11,15,18,23,24,28,29,30].

### 4.7. Strengths and Limitations

Strengths include the four-year age- and sex-stratified description of elevated-HbA1c records, transparent reporting of patient-year rather than unique-patient counts, use of biomarker-specific denominators, exact tests for sparse sex comparisons, and multiplicity adjustment. The revision also aligns the statistical methods with the analyses that are actually reported.

Several limitations materially constrain inference. The annual dataset lacks the total number tested in each age–sex stratum and therefore cannot estimate prevalence. It is record-based and does not support within-person aging or HbA1c trajectories. Detailed biomarker data are available only in adults aged 18–29 years, so cross-age renal, hepatic, and metabolic comparisons cannot be performed. Individual biomarkers were available for only 11–17 of the 49 participants, creating wide confidence intervals and possible selective-testing bias. The archived analytical summary does not preserve complete paired continuous HbA1c and biomarker values, preventing HbA1c-category comparisons, correlation analyses, or multivariable regression. Cross-domain patient-level pairing was also unavailable, so a multisystem burden score cannot be calculated. The combined creatinine/eGFR category prevents separate evaluation of filtration and precludes identification of NA-DKD or calculation of the SUA/sCr. Finally, single abnormal UACR or eGFR measurements cannot establish chronic kidney disease, and relevant confounders such as diabetes duration, adiposity, blood pressure, medications, fasting status, alcohol exposure, and viral-hepatitis status were not consistently represented in the summarized analytical file.

### 4.8. Future Directions

A prospective or fully patient-linked retrospective study should preserve numerical HbA1c and organ biomarkers for all age groups and predefined HbA1c categories. This would permit direct comparison of renal, hepatic, and metabolic parameters across glycemic strata, age-by-HbA1c and sex-by-HbA1c interaction testing, and multivariable adjustment for clinical confounders. Such designs would also align with current recommendations emphasizing repeated patient-level kidney and cardiometabolic risk assessment [10,11,12,13,14,15].

Renal follow-up should retain separate UACR, serum creatinine, and eGFR values and should classify albuminuric and non-albuminuric DKD phenotypes. Where feasible, the SUA/sCr and selected tubular or vascular markers could be evaluated alongside conventional measures to determine whether they improve risk stratification beyond the UACR and eGFR alone [19].

## 5. Conclusions

The study identifies two distinct descriptive patterns: a changing age and sex composition of elevated-HbA1c patient-year records from 2019–2022 and a heterogeneous multiorgan biomarker-abnormality profile in a separate 2022 young-adult subcohort. In that subcohort, total cholesterol, triglycerides, the UACR, urea, uric acid, and ALT were the most frequent abnormalities, whereas exploratory sex differences were imprecise and did not survive false-discovery-rate correction.

The available data do not support an HbA1c dose–response relationship, cross-age biomarker comparison, or causal interpretation because paired continuous HbA1c–biomarker values and older-age biomarker data were not retained in the archived analytical summary. The principal clinical message is therefore one of integrated assessment rather than an HbA1c-centered causal model: renal risk should consider both the UACR and eGFR, including the possibility of non-albuminuric DKD, while lipid and hepatic abnormalities require domain-specific evaluation. Future patient-level studies across age and HbA1c strata are needed to test these relationships directly.

## Figures and Tables

**Table 1:** Annual distribution of elevated-HbA1c records by age and sex, 2019–2022.

Year	Age Group	Male, n	Female, n	Total, n	Share of Annual Records
2019	18–29 years	28	21	49	16.3%
2019	30–59 years	99	32	131	43.7%
2019	≥60 years	71	49	120	40.0%
2020	18–29 years	15	10	25	8.7%
2020	30–59 years	67	78	145	50.2%
2020	≥60 years	60	59	119	41.2%
2021	18–29 years	25	10	35	11.3%
2021	30–59 years	77	82	159	51.5%
2021	≥60 years	60	55	115	37.2%
2022	18–29 years	29	27	56	21.4%
2022	30–59 years	42	33	75	28.6%
2022	≥60 years	79	52	131	50.0%

**Table 2:** Biomarker-abnormality profile among adults aged 18–29 years in 2022.

Biomarker	Males Abnormal, n/N (%)	Females Abnormal, n/N (%)	Overall Abnormal, n/N (%)
UACR	2/5 (40.0%)	3/6 (50.0%)	5/11 (45.5%)
Urea	1/8 (12.5%)	5/7 (71.4%)	6/15 (40.0%)
Creatinine/eGFR classification	0/8 (0.0%)	0/7 (0.0%)	0/15 (0.0%)
Uric acid	2/8 (25.0%)	4/7 (57.1%)	6/15 (40.0%)
Total bilirubin	1/6 (16.7%)	0/5 (0.0%)	1/11 (9.1%)
Alkaline phosphatase	0/8 (0.0%)	0/5 (0.0%)	0/13 (0.0%)
AST	2/8 (25.0%)	0/5 (0.0%)	2/13 (15.4%)
ALT	1/8 (12.5%)	5/9 (55.6%)	6/17 (35.3%)
GGT	0/8 (0.0%)	0/5 (0.0%)	0/13 (0.0%)
Albumin	0/8 (0.0%)	0/5 (0.0%)	0/13 (0.0%)
Total protein	0/8 (0.0%)	0/5 (0.0%)	0/13 (0.0%)
Total cholesterol	6/7 (85.7%)	3/8 (37.5%)	9/15 (60.0%)
Triglycerides	5/6 (83.3%)	2/7 (28.6%)	7/13 (53.8%)
LDL-C	0/6 (0.0%)	2/7 (28.6%)	2/13 (15.4%)
HDL-C	0/6 (0.0%)	0/7 (0.0%)	0/13 (0.0%)
VLDL-C	0/6 (0.0%)	0/7 (0.0%)	0/13 (0.0%)

**Table 3:** Exploratory sex-associated biomarker abnormalities among adults aged 18–29 years.

Biomarker	Female vs. Male OR	95% CI	Fisher *p*	FDR q	φ Coefficient
UACR	1.50	0.14–16.54	1.000	1.000	0.100
Urea	17.50	1.22–250.37	0.041	0.295	0.600
Uric acid	4.00	0.45–35.79	0.315	0.566	0.327
Total bilirubin	0.33	0.01–10.11	1.000	1.000	−0.289
AST	0.24	0.01–6.05	0.487	0.626	−0.337
ALT	8.75	0.74–103.83	0.131	0.295	0.450
Total cholesterol	0.10	0.01–1.29	0.119	0.295	−0.491
Triglycerides	0.08	0.01–1.19	0.103	0.295	−0.548
LDL-C	5.91	0.23–151.15	0.462	0.626	0.395

## Data Availability

The data supporting the findings of this study are available from the corresponding author upon reasonable request and subject to institutional and ethical restrictions.

## Abbreviations

The following abbreviations are used in this manuscript:

AGE	advanced glycation end product
ALP	alkaline phosphatase
ALT	alanine aminotransferase
AST	aspartate aminotransferase
CI	confidence interval
CKD	chronic kidney disease
eGFR	estimated glomerular filtration rate
FDR	false-discovery rate
GGT	gamma-glutamyl transferase
HbA1c	glycated hemoglobin
HDL-C	high-density lipoprotein cholesterol
IRB	Institutional Review Board
LDL-C	low-density lipoprotein cholesterol
MASLD	metabolic dysfunction-associated steatotic liver disease
NF-κB	nuclear factor kappa B
OR	odds ratio
RAGE	receptor for advanced glycation end products
SGLT2	sodium–glucose cotransporter 2
UACR	urine albumin-to-creatinine ratio
VLDL-C	very-low-density lipoprotein cholesterol
DKD	diabetic kidney disease
NA-DKD	non-albuminuric diabetic kidney disease
SUA/sCr	serum uric acid-to-creatinine ratio
